# Exploring the impact of non-emergency inter-hospital neonatal transport on infants and their parents: a mixed-methods systematic review

**DOI:** 10.1136/bmjpo-2026-004846

**Published:** 2026-10-28

**Authors:** Lizanne A Derks, Myrthe L J Partouns, Margreet R Koolen-De Koninck, Anna J Apperloo, Sylvia A Obermann-Borst, Angela M Tjiam, H Rob Taal, Josephine H L Wagenaar

**Affiliations:** 1 Department of Ophthalmology, Erasmus MC, Rotterdam, The Netherlands; 2 Department of Neonatal and Paediatric Intensive Care, Division of Neonatology, Erasmus MC Sophia, Rotterdam, The Netherlands; 3 Care4Neo, Neonatal Patient and Parent Advocacy Organisation, Rotterdam, The Netherlands

**Keywords:** Intensive Care Units, Neonatal, Neonatology, Health services research, Infant

## Abstract

**Background:**

Reliance on inter-hospital neonatal transport is increasing due to regionalisation, centralisation and capacity pressures within neonatal care. Although the effects of transporting critically ill infants are well documented, the impact of non-emergency transfers involving stable infants remains underexplored. The objective of this review was to examine the current literature on how non-emergency inter-hospital transfers affect infants and their parents.

**Methods:**

For this review, Medline, CINAHL and Embase databases were searched for articles that studied neonatal non-emergency transport and its impact on infants’ health or their parents’ well-being. Articles were screened independently by at least two authors; relevant articles were selected through unanimous vote or by consensus reached through team meetings.

**Results:**

Of the 2811 articles screened, 23 studies met the inclusion criteria. 10 studies focused on infant health outcomes, and 13 studies on parental well-being outcomes. Some studies report physiological deterioration following non-emergency inter-hospital transport, particularly when the duration of transport is longer. Available long-term data do not show increased morbidity or mortality among infants who undergo non-emergency transport. Qualitative and quantitative studies reported parental stress and anxiety during transport; however, timely communication, thorough preparation, ongoing reassurance and support for active parental involvement may contribute to more positive experiences.

**Conclusion:**

Although this review is limited by substantial heterogeneity in outcome reporting, predominantly unadjusted analyses and findings that were frequently derived from single studies, the available evidence does not demonstrate consistent adverse effects of non-emergency neonatal transport. In contrast, negative parental experiences related to transport were frequently reported. Establishing the level of clinical stability required for safe non-emergency transport should be a priority, alongside implementing interventions that support parental well-being throughout the transport process.

**WHAT IS ALREADY KNOWN ON THIS TOPIC**

- Inter-hospital neonatal transport is increasingly used to move infants between levels of care within regionalised and centralised neonatal care systems.
- While the effects of transporting critically ill infants are well documented, less is known about the consequences of transporting stable infants.

**WHAT THIS STUDY ADDS**

- Non-emergency transport was not consistently associated with adverse long-term clinical outcomes in infants, although short-term physiological deterioration has been reported, particularly with increasing duration of transport.
- Reported negative parental experiences related to transport emphasise the need for communication, preparation and support from healthcare providers.

**HOW THIS STUDY MIGHT AFFECT RESEARCH, PRACTICE OR POLICY**

- Because non-emergency transport allows for planned decision-making, there is an opportunity to establish clear transport criteria that ensure transfers do not compromise infant health. In addition, these findings highlight the need for interventions that help healthcare providers prepare parents for transfer, as even non-emergency transport can involve unexpected changes that require clear communication, anticipatory guidance and emotional support.

## Introduction

 In many high-income countries, care for sick or preterm neonates is delivered within a regionalised system, comprised of four levels of care ranging from basic care units (level I) to neonatal intensive care units (NICUs) for the most critically ill neonates (level IV). Level III or IV neonatal care is generally concentrated in selected hospitals, typically academic or tertiary referral centres, while level I or II neonatal care is also available in regional or community hospitals.^1 2^ To ensure infants receive the level of care appropriate to their needs, infants may be transferred between hospitals during their admission.^1^

Neonatal transport can vary in purpose and in level of urgency. Emergency neonatal transport involves critically ill infants who require immediate access to a higher level of care, whereas non-emergency transport concerns relatively stable infants who are transferred for convalescence at a lower-level care unit, are scheduled for semi-elective surgical procedures or relocated due to capacity constraints.^1–3^ In many regions, pressure on NICU capacity is increasing due to the growing number of surviving extremely preterm and medically complex infants requiring longer stays, as well as the ongoing regionalisation and centralisation of specialised neonatal services into fewer centres.^4–6^ As a result, back-transfers and other forms of elective, non-emergency transport are commonly performed. Accordingly, paediatricians and other healthcare providers across all different level care settings are regularly involved in preparing infants for transport, continuing their care after arrival and supporting families throughout the transition.

A substantial body of research has examined the impact of neonatal transport, with most studies focusing on emergency transport of critically ill neonates. These studies suggest that, while emergency transport provides the necessary access to specialised care, transported infants may experience physiological stress and deterioration as they are exposed to noise and vibrations exceeding acceptable adult standards during transport.^7–9^ Moreover, neonatal transport has been linked to an increased risk of neurodevelopmental impairment at 3 years of age.^10^ In addition to these clinical concerns, parents often have negative perceptions of transport, citing feelings of distress, anxiety and loss of control.^11 12^

However, the potential risks and benefits of non-emergency neonatal transport of relatively stable infants are less well defined and are often not clearly differentiated from those of other forms of transport. Non-emergency transport may still destabilise the infant’s condition or disrupt continuity of care. At the same time, given the non-urgent nature of these transfers, healthcare professionals have the opportunity to carefully weigh possible advantages and disadvantages of transporting these infants, find the optimal timing of transport and ensure that infants, families and the receiving care teams are adequately prepared. Understanding the impact of non-emergency transport is therefore relevant to healthcare providers across all different levels of neonatal care, as such knowledge may inform both transferring and receiving teams about ways to improve transport safety, patient handover, convalescent care and parental support during the transition between hospitals. Accordingly, we conducted a systematic review of quantitative and qualitative studies to examine how non-emergency inter-hospital neonatal transport impacts both infant health and parental well-being.

## Methods

### Design

This review was conducted and reported following the JBI guidance for mixed-methods systematic reviews^13^ and the PRISMA (Preferred Reporting Items for Systematic reviews and Meta-Analyses) statement for systematic reviews.^14^ Where applicable, we used the eMERGe guidance for reporting qualitative synthesis.^15^ Our protocol was published as a pre-print on medRxiv, available at https://doi.org/10.1101/2025.03.19.25322589. Initially, we aimed to conduct a scoping review to explore the evidence on non-emergency transport. During screening, however, we identified sufficient eligible studies to deviate from this protocol and conduct a systematic review with mixed-methods synthesis.

### Research question and eligibility criteria

We aimed to synthesise the existing evidence on how non-emergency inter-hospital transport affects infants and their parents. Eligibility criteria, defined using the Participants Concept Context framework,^16^ were: (1) the study must include a population of hospital-admitted infants and/or their parents or caregivers; (2) the study intervention must involve (majority) non-emergency, inter-hospital transport of these infants and (3) the study must be empirical research reporting on infant health or parental well-being. For this review, non-emergency inter-hospital transport was defined as any transport of a clinically stable infant between two hospitals or hospital facilities at least 72 hours after birth. No restrictions were applied to outcome measures or comparator groups, provided that the effects of transport on infants and/or parents were described. Full-text publications in Dutch or English were eligible, with no limits on year of publication or country of origin.

### Information sources and search strategy

The search strategy was developed in collaboration with a biomedical information specialist from the Erasmus MC Medical Library. The search consisted of terms related to (preterm) infants and transports between hospitals (online supplemental table S1). Medline, Embase and CINAHL were searched until 17 November 2025. In addition to the database searches, backward and forward snowballing was conducted manually to identify potentially relevant papers in the reference or citation lists of included studies.

### Study selection

Identified publications were independently screened for eligibility by five authors (LAD, MLJP, MRK-DK, AJA, JHLW) using Covidence (Veritas Health Innovation, 2023). Decisions required two unanimous votes; conflicts were resolved in consensus meetings in the presence of all screening authors. Full-text examination was performed by four authors (LAD, MLJP, MRK-DK, JHLW) and followed the same consensus procedures as initial screening, with the addition of recording the reasons for excluding publications. If multiple reasons for exclusion applied, the first applicable study characteristic following the PICO (Population, Intervention, Comparison, Outcome) order was noted. Inter-rater reliability was estimated using Cohen’s κ and percentage agreement per screening author pair.

### Data extraction and quality appraisal

A data charting form was developed, reviewed and piloted in Covidence. The form collated study characteristics, baseline population characteristics (infants and/or parents), transport characteristics, outcome measures and key findings.

For quantitative studies, extracted data included reported outcome measures, direction of findings and relevant statistical information where available. For qualitative studies, extracted data included participant quotes (first order constructs) and primary author interpretations (second order constructs). Risk of bias was assessed using the Mixed Methods Appraisal Tool (MMAT).^17^

Data extraction and quality assessment were performed by three authors (LAD, MLJP, MRK-DK). For each study, qualitative and/or quantitative data extraction and quality assessment were conducted independently by at least two authors, with subsequent verification by at least one author. Any discrepancies were resolved through collective discussion until consensus was reached. No studies were excluded based on risk of bias; however, identified limitations were considered during the interpretation of study findings.

### Data synthesis and reporting

Study characteristics, baseline population characteristics and transport characteristics were summarised in descriptive tables. Outcome measures and key findings were grouped into two domains: infant health outcomes and parental well-being outcomes. Within these domains, we analysed the quantitative and qualitative evidence separately using methods appropriate to each evidence type before integrating the findings (convergent segregated synthesis).^13^

Quantitative findings were tabulated and grouped by outcome measure. Due to heterogeneity in study designs, populations and outcome definitions, findings were synthesised narratively. Where appropriate, patterns in the direction and consistency of effects were identified across studies. Meta-analysis and formal assessment of outcome-level certainty of evidence were not feasible due to heterogeneity in outcome reporting. We assessed reporting bias by examining whether studies reported all outcomes described in their methods; formal analysis of selective non-reporting was not feasible due to heterogeneity in outcome reporting.

Qualitative findings were synthesised using the principles of meta-ethnography, which facilitate comparison of qualitative findings across studies and the development of an overarching interpretation of the data.^18 19^ The first-order and second-order constructs extracted from individual studies were entered into a Microsoft Excel sheet for structured comparison. Translation of the constructs across studies was led by one author (LAD) and was conducted using an inductive approach. To identify overlapping findings across studies, extracted constructs were first paraphrased and compared, followed by iterative reading and re-reading to ensure that the original meaning of each construct was accurately represented. The paraphrased and combined constructs were subsequently grouped into domains where appropriate, and the resulting grid was reviewed and discussed with the other authors to ensure agreement and coherence. To formulate higher-order interpretations, conceptually overlapping constructs were first consolidated into key concepts through iterative reciprocal and refutational translation, that is, clustering similar and/or contradicting concepts. Relationships between concepts were then examined and synthesised into interpretive statements, which were subsequently integrated into a final line-of-argument representing the overarching interpretation of parental experiences across studies. The process of higher-order synthesis was led by one author (LAD), and syntheses were reviewed by the other authors to ensure coherence and to consider potential alternative interpretations.

Interpretive bias was minimised through independent data extraction, iterative discussion and consideration of alternative interpretations at multiple stages of the qualitative analysis. Reflexivity was supported by the multidisciplinary composition of the research team, as the team of authors consisted of researchers from various professional backgrounds, including clinicians (neonatologist, paediatric ophthalmologist) and (research) nurses involved with neonatal transport, PhD and medical students acquainted with the subject, and a representative of a neonatal parent and patient advocacy organisation.

### Ethics and patient and public involvement statement

Due to the non-interventional nature of this research, no ethics review was required for this review. A scientific expert from the Dutch neonatal parent and patient advocacy organisation *Care4Neo* was involved in the interpretation of the results and writing of the manuscript (SAO-B).

## Results

### Study inclusion and characteristics

Figure 1 summarises the inclusion process. After deduplication, the search strategy and snowballing yielded 2811 publications, of which 23 were included.^20–42^ Two included publications reported separate components of the same study.^27 35^ Inter-rater agreement among screening author pairs varied between 40.0%–89.2% (κ−0.04-0.60) and 91.5%–100.0% (κ=0.48-1.00) during title and abstract screening and full-text screening, respectively (online supplemental table S2). Table 1 provides an overview of study characteristics. Most studies were published within the last 15 years (n=15, 65.2%), and originated from North America (n=11, 47.8%) and Europe (n=10, 43.5%). 10 studies (43.5%) assessed infant health and 13 studies (56.5%) assessed parental well-being. The number of transported participants varied widely across studies, ranging from 6 to 504, with fewer than 100 participants in over half of the studies (n=14, 60.9%). In most studies (n=20, 87.0%), the indication for transport was back-transport, referring to previously critically ill infants returning from a higher-level unit to their local lower-level unit.

**Figure 1:**
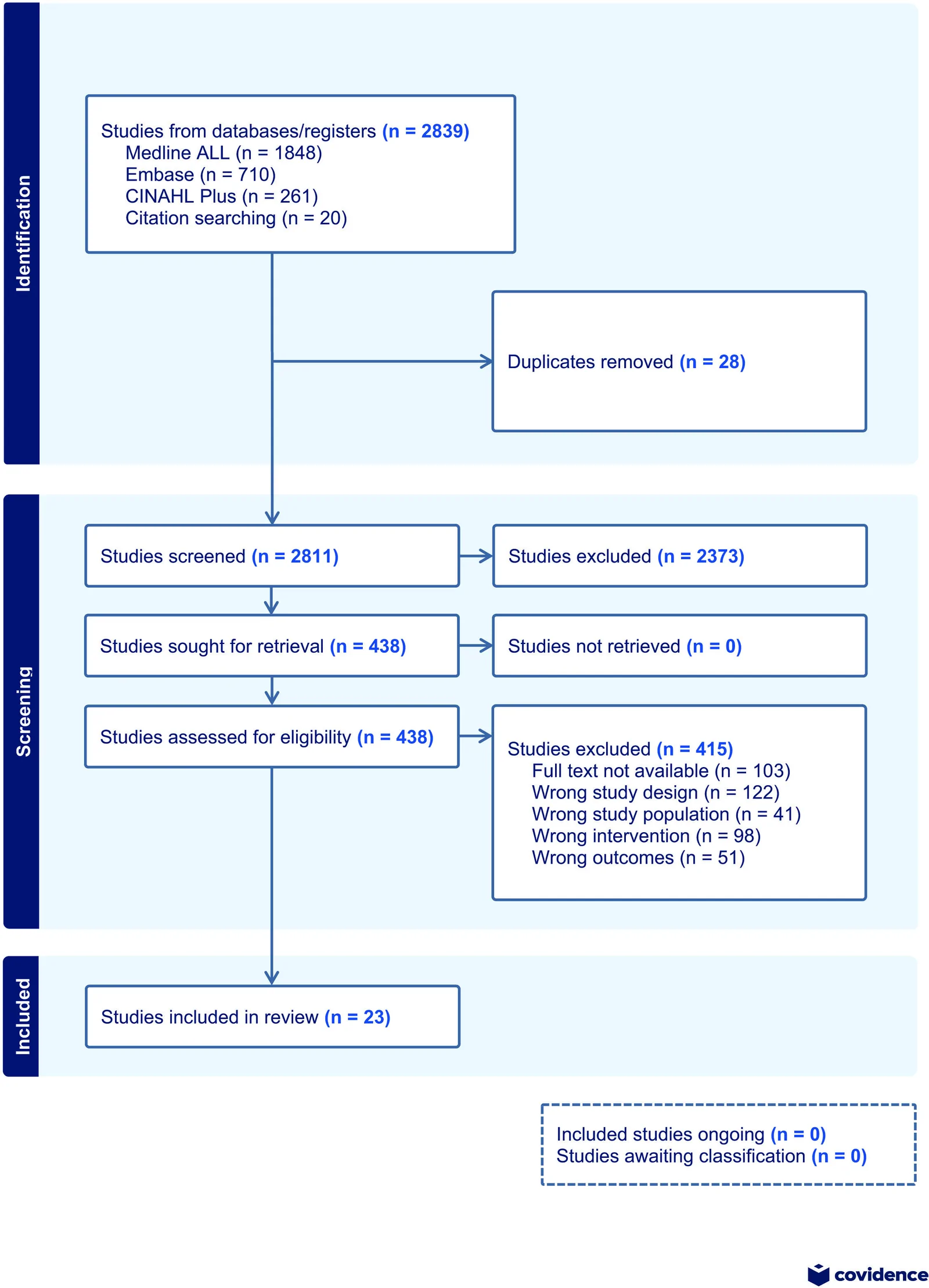
Study inclusion flow chart. Flow chart summarising the study inclusion process. CINAHL, Cumulative Index to Nursing and Allied Health Literature; MEDLINE, Medical Literature Analysis and Retrieval System Online.

**Table 1:** Study background characteristics

	Infant health, n (%)	Parental experience, n (%)
Number of studies	10 (100)	13 (100)
Publication year		
<2000	1 (10.0)	3 (23.1)
2000–2009	2 (20.0)	2 (15.4)
2010–2019	5 (50.0)	4 (30.8)
>2019	2 (20.0)	4 (30.8)
Country		
Belgium	1 (10.0)	0 (0.0)
Canada	0 (0.0)	3 (23.1)
India	1 (10.0)	0 (0.0)
Netherlands	1 (10.0)	2 (15.4)
New Zealand	0 (0.0)	1 (7.7)
Norway	0 (0.0)	1 (7.7)
Sweden	2 (20.0)	1 (7.7)
Switzerland	1 (10.0)	0 (0.0)
UK	0 (0.0)	1 (7.7)
USA	4 (40.0)	4 (30.8)
Study methodology		
Quantitative non-randomised	10 (100.0)	1 (7.7)
Before-and-after study	6 (60.0)	0 (0.0)
Cohort study	4 (40.0)	0 (0.0)
Cross-sectional analytic study	0 (0.0)	1 (7.7)
Quantitative descriptive (survey)	0 (0.0)	2 (15.4)
Mixed-methods	0 (0.0)	4 (30.8)
Qualitative	0 (0.0)	6 (46.2)
Phenomenology	0 (0.0)	1 (7.7)
Qualitative description	0 (0.0)	5 (38.5)
Number of transported study participants		
<50	3 (30.0)	8 (61.5)
50–100	3 (30.0)	0 (0.0)
100–200	2 (20.0)	4 (30.8)
200–400	1 (10.0)	1 (7.7)
>400	1 (10.0)	0 (0.0)
Population		
Preterm and term	6 (60.0)	7 (53.8)
Preterm <37 weeks	2 (20.0)	4 (30.8)
Preterm <29 weeks	0 (0.0)	1 (7.7)
VLBW	1 (10.0)	1 (7.7)
ELBW	1 (10.0)	0 (0.0)
Mode of transport		
Ground ambulance	9 (90.0)	13 (100)
Consecutive air and ground ambulance	1 (10.0)	0 (0.0)
Indication for transport		
Back-transport	7 (70.0)	13 (100)
Specialist surgery, treatment or evaluation and/or convalescent care	2 (20.0)	0 (0.0)
Not described	1 (10.0)	0 (0.0)
Comparator		
None	0 (0.0)	11 (84.6)
Non-transported infants*	4 (40.0)	1 (7.7)
Pre-transport state	6 (60.0)	0 (0.0)
Transport with parental preparation model	0 (0.0)	1 (7.7)

* Direct discharge from initial care centre to the home environment.

ELBW, extremely low birth weight <1000 g; VLBW, very low birth weight <1500 g.

### Mixed Methods Appraisal Tool

Table 2 summarises quality assessment for included quantitative non-randomised studies (n=11),^20–22 24 31–35 39 40^ quantitative descriptive studies (n=2),^36 38^ mixed-methods studies (n=4)^21 23 26 41^ and qualitative studies (n=6).^27–30 37 42^ In quantitative non-randomised studies, potential bias mainly stemmed from missing or unclear confounder adjustment (73%). Additionally, although not assessed in the MMAT, these studies often involved small and/or heterogeneous populations (online supplemental table S3). Both quantitative descriptive studies showed high risk of nonresponse bias.^36 38^ For mixed-methods studies, any risk of bias was confined to two out of four studies.^25 41^ Qualitative studies were generally low-risk, though there were unclarities in (the interpretation of) the results for some studies.^29 30 37 42^ While not specifically questioned in the MMAT, three qualitative studies^28 30 37^ may be subject to recall bias, as parental well-being was assessed months to years post-transport. We did not identify concerns related to selective non-reporting.

**Table 2:** Risk of bias assessment using the Mixed Methods Appraisal Tool

Study design, author	Quality assessment criteria				
	Q1	Q2	Q3	Q4	Q5
Quantitative non-randomised					
Arora *et al*^20^	Low	Low	Low	Low	Low
Attar *et al*^21^	Low	Low	Low	High	Unclear
Chakraborty *et al*^22^	Low	Low	Low	High	Low
de Preud’homme d’Hailly de Nieuport *et al*^24^	Unclear	Low	Unclear	High	Low
Hennequin *et al*^31^	Low	Low	Low	High	Low
Karlsson *et al*^32^	Low	Unclear	High	Unclear	Low
Lainwala *et al*^33^	Low	Low	High	Low	Low
Lynch *et al*^34^	Low	Unclear	Low	High	Low
Van den Berg *et al*^39^	Unclear	Low	High	High	Unclear
Zwissig *et al*^40^	High	Low	Low	High	Low
Murthy *et al*^35^	High	Low	High	Low	Unclear
Mixed methods					
Donohue *et al*^26^	Low	Low	Low	Low	Low
De Bijl-Marcus *et al*^23^	Low	Low	Low	Low	Low
Dodds-Azzopardi and Chapman^25^	Unclear	Low	Low	Unclear	High
Chant *et al*^41^	Unclear	Unclear	Low	Unclear	Low
Qualitative					
Helder *et al*^30^	Low	Low	Unclear	Unclear	Low
Dosani *et al*^27^	Low	Low	Low	Low	Low
Granrud *et al*^28^	Low	Low	Low	Low	Low
Hanrahan *et al*^29^	Low	Low	Unclear	Low	Low
Sommer and Cook^37^	Low	Low	Unclear	Low	Low
Kuhnly and Freston^42^	Low	Low	Low	Unclear	Low
Quantitative descriptive					
Van den Berg and Lindh^38^	Low	Low	Low	High	Low
Slattery *et al*^36^	Low	Unclear	Low	High	Low

Quantitative non-randomised studies:

Q1: Are the participants representative of the target population?

Q2: Are measurements appropriate regarding both the outcome and intervention (or exposure)?

Q3: Are there complete outcome data?

Q4: Are the confounders accounted for in the design and analysis?

Q5: During the study period, is the intervention administered (or exposure occurred) as intended?

Mixed-methods studies:

Q1: Is there an adequate rationale for using a mixed-methods design to address the research question?

Q2: Are the different components of the study effectively integrated to answer the research question?

Q3: Are the outputs of the integration of qualitative and quantitative components adequately interpreted?

Q4: Are divergences and inconsistencies between quantitative and qualitative results adequately addressed?

Q5: Do the different components of the study adhere to the quality criteria of each tradition of the methods involved?

Qualitative studies:

Q1: Is the qualitative approach appropriate to answer the research question?

Q2: Are the qualitative data collection methods adequate to address the research question?

Q3: Are the findings adequately derived from the data?

Q4: Is the interpretation of results sufficiently substantiated by data?

Q5: Is there coherence between qualitative data sources, collection, analysis and interpretation?

Quantitative descriptive studies:

Q1: Is the sampling strategy relevant to address the research question?

Q2: Is the sample representative of the target population?

Q3: Are the measurements appropriate?

Q4: Is the risk of nonresponse bias low?

Q5: Is the statistical analysis appropriate to answer the research question?

### Impact of transport on infant health

#### Study design and outcome measures

Across included studies, infant health outcomes were assessed using two overarching approaches: (1) comparing the pre-transport and post-transport clinical state of the infant (before-and-after studies, n=6)^20 24 31 32 39 40^ and (2) comparing outcomes of transported infants to those of non-transported infants (cohort studies, n=4),^21 22 33 34^ see table 1. Specifications of infant health outcome measures are listed in online supplemental table S4. Two studies also described overall incidences of adverse events without comparative analysis.^24 31^

#### Infant characteristics

Online supplemental table S3 details infant characteristics. Across studies, gestational age (GA) was the only infant characteristic consistently reported, with mean, median or majority GA ranging from 26 to 36 weeks. Based on available data, included infants were relatively clinically stable, with mean or median postnatal age ranging from 17 to 57 days, current weight above 1300 g and predominantly no or minimal respiratory support at the time of transport.

#### Comparing pre-transport and post-transport clinical state

Across included before-and-after studies, findings on changes in infants’ physiological stability after transport were variable, with some studies finding significant deterioration and others finding stable or even improving physiological parameters. Two before-and-after studies report evidence of physiological deterioration after transport, including an increased Transport Risk Index of Physiologic Stability (TRIPS) score (54% of transported infants),^20^ increased oxygen requirement (before 22.8% SD 10.0; after 24.5% SD 10.6; p=0.02),^24^ decreased body temperature (before 37.1°C SD 0.4; after 36.7°C SD 0.5; p<0.001),^24^ and higher incidence of bradycardia episodes (before 1.1 SD 1.8; after 2.9 SD 4.0; p<0.001)^24^ (table 3). Within the respective studies, longer transport duration was reported to be associated with a significantly larger decrease in body temperature^24^ (30 min −0.2°C SD 0.6; 60 min −0.4°C SD 0.6; p=0.046) and a significantly higher incidence of an increased TRIPS score^20^ (≤15 min 45%; >15 min 65%; p=0.03) after transport. In contrast, several studies reported comparable pre-transport and post-transport values for respiratory rate, oxygen saturation, heart rate, body temperature and various pain scores, although statistical testing was not described.^31 39 40^ One study reported increasing heart rate variability (HRV) during ground and air transport, suggesting stabilisation.^32^

**Table 3:** Infant health outcomes

Outcome category	Characteristics		
Pre-transport versus post-transport physical state	No. of transported infants (studies)	Effect summary	P value
Respiratory rate	138 (3 studies)	Descriptive only^31 39 40^	N/A
Respiratory support type	91 (1 study)	No change^24^	0.12
Oxygen requirement	91 (1 study)	Deterioration^24^	0.02
Oxygen saturation	138 (3 studies)	Descriptive only^31 39 40^	N/A
Heart rate	154 (4 studies)	No change^32^	>0.05
		Descriptive only^31 39 40^	N/A
Heart rate variability	16 (1 study)	Improvement^32^	<0.05
Bradycardia episodes	91 (1 study)	Deterioration^24^	<0.001
Body temperature	209 (3 studies)	Descriptive only^31 39^	N/A
		Deterioration^24^	<0.001
ALPS	24 (1 study)	Descriptive only^39^	N/A
PIPP-R	20 (1 study)	Descriptive only^40^	N/A
CB	20 (1 study)	Descriptive only^40^	N/A
Salivary cortisol levels	20 (1 study)	No change^40^	0.663

Transported versus non-transported infants	No. of transported infants (studies), No. of controls	Effect summary	P value
Apnoea episodes	55 (1 study), 58	No difference^34^	>0.05
Bradycardia episodes	55 (1 study), 58	No difference^34^	>0.05
Oxygen requirement	55 (1 study), 58	No difference^34^	>0.05
Feeding intolerance	55 (1 study), 58	No difference^34^	>0.05
Daily weight gain	55 (1 study), 58	Favours transport^34^	<0.05
New clinical problems	55 (1 study), 58	Descriptive only^34^	N/A
Return to higher-level unit	55 (1 study), 58	Descriptive only^34^	N/A
Mortality	705 (2 studies), 4447	No difference^22^	>0.05
		Favours no transport^33^	0.04
Length of stay	608 (2 studies), 4509	Favours transport^33^	<0.0001
		Favours no transport^21^	<0.0001
Readmissions	809 (3 studies), 4564	No difference^21 22^	>0.05
		Favours no transport^33^	0.02
Emergency room visits	201 (1 study), 55	No difference^22^	>0.05
Outpatient visits	201 (1 study), 55	No difference^22^	>0.05
Weight	504 (1 study), 4392	No difference^33^	0.5
Height	504 (1 study), 4392	No difference^33^	0.9
Head circumference	504 (1 study), 4392	No difference^33^	0.4
MDI	504 (1 study), 4392	Favours transport^33^	0.01
PDI	504 (1 study), 4392	No difference^33^	0.08
Blindness	504 (1 study), 4392	No difference^33^	0.8
Hearing impairment	504 (1 study), 4392	No difference^33^	0.3
Cerebral palsy	504 (1 study), 4392	No difference^33^	0.999
NDI	504 (1 study), 4392	Favours transport^33^	0.03

Effect summary indicates whether outcomes significantly improved, remained unchanged or worsened after transport, or whether findings significantly favoured transport, favoured no transport or showed no clear difference between transported and non-transported groups.

NDI was defined as presence of one or more of the following: Mental Developmental Index (MDI) 70, Psychomotor Developmental Index (PDI) 70, cerebral palsy, hearing impairment or blindness.

ALPS, Astrid Lindgren Children’s Hospital Pain Scale; CB, Comfort Behaviour Score; NDI, Neurodevelopmental Impairment; PIPP-R, Premature Infant Pain Profile-Revised.

#### Comparing transported and non-transported infants

Studies comparing transported and non-transported infants reported varying results across outcomes, with most studies showing no significant differences between groups, and some reporting better outcomes for either transported or non-transported infants (table 3).

Mortality, readmission rates and length of stay (LOS) were each assessed in multiple studies, with conflicting findings in each case. One study found lower mortality and readmission rates at 18–22 months corrected age (CA) among non-transported infants within a population of extremely low birth weight (ELBW) infants; however, after adjustment for neonatal and demographic characteristics, only readmission rates remained significantly lower for non-transported infants.^33^ In contrast, two studies including mixed populations of preterm and term infants both found no differences in mortality and readmission rates between transported and non-transported infants, although these outcomes were assessed over shorter follow-up periods (online supplemental table S4).^21 22^ Regarding the effect of transport on LOS, longer stays were reported for non-transported infants in the ELBW cohort,^33^ while longer stays were reported among transported infants in a mixed cohort.^21^

Regarding developmental outcomes, significantly better motor and neurodevelopmental indices (Mental Developmental Index and Neurodevelopmental Impairment) at 18–22 months CA were reported among transported infants in the ELBW cohort; however, after adjustment for neonatal and demographic characteristics, these differences were no longer significant.^33^ In another study, daily weight gain measured up until discharge was higher among transported preterm and term infants compared with non-transported controls, thus favouring transport.^34^ Moreover, this study found comparable rates for new clinical problems during convalescence (online supplemental table S4) between back-transported infants and non-transported controls (0.080 vs 0.084 per day) and found that 7.2% of back-transferred infants required readmission to a higher-level centre, while 14% of non-transported controls were reassigned to higher-level care within the same facility.^34^

For the remaining outcomes assessed in these studies, no differences between transported and non-transported infants were found. Reported outcomes included apnoea, bradycardia, oxygen requirement and feeding intolerance during convalescence,^34^ emergency department or outpatient visits,^22^ growth outcomes (weight, height and head circumference)^33^ and developmental outcomes (Psychomotor Developmental Index, blindness, hearing impairment and cerebral palsy).^33^

#### Adverse events during and after transport

Two studies reporting adverse events both described events occurring during and after transport, with variation in the type and frequency of reported events. One study reported a 14% (13 out of 91) rate of suspected late-onset sepsis within a week of transport, of which 46% was confirmed as bacterial sepsis.^24^ Another study reported a 14% rate (3 out of 22) of medical adverse events during transport, including one instance of increased oxygen requirement, one instance of low temperature at baseline and one instance of severe deterioration of the infant in the ambulance requiring the ambulance to stop.^39^

### Impact of transport on parents’ well-being

#### Study design and outcome measures

Studies on parental well-being employed varying study designs, including quantitative (n=3),^35 36 38^ qualitative (n=6)^27–30 37 42^ and mixed-methods designs (n=4),^23 25 26 41^ and varying methods, including interviews (n=6),^25 26 28 30 37 42^ surveys (n=5),^27 35 36 38 41^ focus groups (n=1)^29^ or a combination of these tools (n=1).^23^ Interviews and focus group discussions occurred at varying intervals in relation to the child’s admission: directly after transport,^26^ within a week of transport,^23 25 42^ within months^28 30^ or years^37^ after transport, or at an unclear interval.^29^ Surveys were mailed or given to parents during admission^41^ or after discharge.^27 35 36 38^ Studies that quantified parental well-being in relation to neonatal transport used various measures, including stress, perceived quality of care, satisfaction with transfer and readiness for discharge. In addition, associations between these outcomes and specific aspects of care were examined. Assessment tools included Likert scales, Visual Analogue Scales (VAS) or other instruments (table 4).

**Table 4:** Quantitative parental well-being outcomes

Outcome	First author	Variable	Positive impact	No impact	Negative impact	P value
Stress	Dodds-Azzopardi and Chapman^25^	Lower level of education	✔			0.002
		Lower level of occupation	✔			0.01
		Lower social position	✔			0.005
		Lower income	✔			0.0003
	Slattery *et al*^36^	Infant problems after transfer			✔	0.0231
		Longer length of stay	✔			0.0101
Likert scales on the NICU-TQS	Slattery *et al*^36^	Difference in physician practice			✔	0.0138
		Infant problems after transfer			✔	0.0069
Likert scales on the CH-TQS	Slattery *et al*^36^	Difference in nursing practice			✔	<0.0001
		Difference in physician practice			✔	<0.0001
		Infant problems after transfer			✔	<0.0001
		Preparation before transfer	✔			<0.0001
Readiness for and satisfaction with discharge	Donohue *et al*^26^	Back-transport		✔		>0.05
VAS for satisfaction surrounding transfer	Van den Berg and Lindh^38^	Prior contact with receiving unit	✔			<0.001
Likert scale for overall satisfaction with transfer	Murthy *et al*^35^	Adequate response to parents’ questions	✔			<0.0001
		Understanding of infant’s condition at receiving hospital	✔			0.0006

CH-TQS, Community Hospital Transfer Quality Scale, representing parents’ experience at the community hospital; NICU, neonatal intensive care unit; NICU-TQS, NICU Transfer Quality Scale, representing parents’ experience at the NICU and during transport; VAS, Visual Analogue Scale.

#### Parent characteristics

Online supplemental table S5 details parent characteristics. Studies primarily included mothers,^25–27 29 30 35–37 41^ though two studies conducted most interviews with both mothers and fathers, either jointly^19^ or individually.^42^ In studies with available demographic data, parents were typically married, white and college-educated, except in the study by Donohue *et al.*, where most parents were non-white, nearly half were single and fewer than one-third had a college degree.^26^

#### Qualitative parental well-being outcomes

Across qualitative and mixed-methods studies, both complementary and contradictory parental experiences related to neonatal transport and convalescent care were reported; while some studies described transport as distressing or unwelcome, others reported more positive experiences. Through cross-study comparison of the extracted first-order and second-order constructs (ie, participant quotes and primary author interpretations, respectively) experiences were grouped into domains according to whether they described transport or convalescent care as positive or negative, and whether they related to parent or infant characteristics, parental perceptions of care or provider behaviours (online supplemental table S6). Based on these constructs, nine key concepts describing parental experiences during neonatal non-emergency transport and convalescent care were identified: (1) geographical accessibility; (2) clinical rationale and impact; (3) communication and information access; (4) continuity and familiarity; (5) fear and psychological stress; (6) routines and relationships; (7) parental involvement, agency and control; (8) environment and unit atmosphere and (9) perceived safety and quality of care (online supplemental table S7). The most common concept, described across all 10 studies with qualitative data, was the importance of adequate communication between healthcare providers and parents and its positive impact on parents’ experiences. Through meta-ethnography, a line of argument was formulated, stating that while parents experience stress and fear during the transport process, parental experiences can be positively impacted through communication, preparation, reassurance and active parental involvement (online supplemental table S7).

#### Quantitative parental well-being outcomes

Parental stress was explicitly assessed in two studies reporting quantitative parental well-being outcomes (table 4). In one study, 77% (34 out of 44) of parents ‘reported stressful incidents surrounding selected aspects of the transfer’.^25^ In another study, mothers rated the ‘overall level of stress related to the transfers of their infants’ with a mean score of 3.13 (SD=1.1), where the score ranged from 1, very stressful, to 5, no stress at all.^36^ These two studies also identified several factors associated with increased or decreased stress levels: lower levels of education, occupation, social position and income, and longer LOS were associated with lower reported stress levels, while infant problems after transfer were associated with higher stress levels.^25 36^

One study reported parents’ overall satisfaction with the transport process, which was rated >5 on a Likert scale from 1 to 10, with 10 being most satisfied, by 64% of parents.^35^ Three studies reporting quantitative parental well-being outcomes identified several factors associated with parents’ ratings of the transport experience.^35 36 38^ In a study focused on mothers’ experiences, differences in physician and nursing practices between units and infant problems after transport were associated with less favourable ratings. Preparation before transport was associated with more favourable ratings.^36^ Two other studies found that prior contact with the receiving unit,^38^ adequate responses to parents’ questions^35^ and understanding of the infant’s condition at the receiving hospital^35^ were associated with more favourable parental ratings.

In a separate study, improved readiness for discharge was not significantly associated with back-transport.^26^

## Discussion

This review examined the impact of non-emergency inter-hospital neonatal transport on infant health outcomes and parental well-being. The available evidence is insufficient to determine whether non-emergency transport is associated with long-term morbidity or mortality. Although adverse long-term effects were not consistently identified, reports of physiological deterioration and transport-related adverse events suggest that potentially clinically relevant effects of current transport strategies cannot be excluded. Within studies that focused on parental well-being, several themes related to parent-reported experiences of non-emergency neonatal transport recurred across both qualitative and quantitative studies. These themes could be broadly categorised as relating to internal factors, such as parents’ emotions and expectations, as well as external factors, such as hospital policies and interactions with healthcare providers. Studies identified communication, preparation, reassurance and opportunities for parental involvement as recurring elements valued by parents and linked to more positive parental experiences during transport.

### Infant health

The available evidence on physiological changes during and after transport suggests that non-emergency transport may be associated with higher TRIPS score, lower body temperature, greater oxygen requirement and more frequent bradycardic episodes. However, these findings are derived from a limited number of before-and-after studies that reported changes either on arrival^20^ or up until 7 days after transport.^24^ While the findings regarding the TRIPS score originate from a study that adjusted for potential confounders,^20^ the study reporting changes in body temperature, oxygen requirement and bradycardia was a short report with a relatively small sample size, which did not provide adjusted analysis. Similarly, another study reported a significant increase in HRV during transport, which could be interpreted as potential indication of improved clinical stability; however, as this finding was unrelated to exposure to sound and vibration during transport, the original authors indicate that it may instead reflect natural stabilisation rather than an effect of transport itself.^32^ The other before-and-after studies included in this review were limited by small sample sizes and reliance on descriptive analyses without statistical comparison of physiological measurements before and after transport.^31 39 40^ As a result, the impact of transport on physiological parameters is difficult to interpret, as causal attribution remains uncertain. Given the lack of adequate adjustment for confounding and control groups, it is not possible to determine whether observed deterioration is attributable to transport itself or to the infant’s underlying clinical course. Moreover, the study demonstrating a significant increase in TRIPS scores included only very low birth weight infants (online supplemental table S3), which limits the generalisability of these findings to a broader population of transported neonates. At the same time, the reported transport-related adverse events indicate that transport is not without potential risk and requires careful clinical consideration.

Comparison of physiological parameters (apnoea, bradycardia, oxygen requirement, feeding intolerance, daily weight gain) between transported and non-transported infants over a longer period of convalescence largely found no differences between groups. Although higher daily weight gain was observed in transported infants in one study, the original authors of the corresponding paper noted that this difference was likely influenced by discharge timing, rather than a true clinical benefit of transport.^34^ Similarly, no consistent associations were found between transport and long-term morbidity, mortality or poorer developmental outcomes.^21 22 33^ The interpretation of these findings is complicated by the fact that several studies comparing transported infants to non-transported controls had smaller sample sizes and did not adjust their findings for differences in baseline infant condition or ongoing clinical management,^21 22 34^ thereby limiting causal inference.

On an organisational level, findings regarding both LOS and readmissions were conflicting,^21 33^ which may be related to differences in study design, case mix and healthcare organisation between studies. Among transported infants, Lainwala *et al* found reduced LOS, but, even after adjustment for neonatal and demographic characteristics, significantly higher readmission rates. Other studies reported an inverse or no association between transport and LOS or readmission rates.^21 22^ Comparison between studies is complicated by limited statistical adjustment and likely heterogeneity in discharge and readmission practices. Moreover, Lainwala *et al* only included ELBW infants (online supplemental table S3), limiting the comparability of their findings within this review and generalisability to broader populations of transported neonates. Consequently, it remains uncertain whether differences in LOS and readmission rates reflect the effects of transport itself, or broader differences in patient populations or care practices after transport and/or discharge.

### Parental well-being

In contrast to the heterogeneity and uncertainty surrounding infant health outcomes, parental distress emerged as a recurring finding across both qualitative and quantitative studies focused on parental well-being. Although non-emergency transport is typically planned for infants who no longer require the highest level of care, themes of stress, anxiety and fear recurred within parent-reported experiences of transport. Aagaard *et al.* have previously noted that while care providers often view back-transport as a routine step when the infant becomes ‘too well’ for their current unit, parents may still perceive their child as critically ill, and, as a result, fear transport.^11^ Two recent publications on neonatal healthcare transitions, including both intra-hospital and inter-hospital transports, also underline that parental experiences of distress, fear and separation are common across transition scenarios, often arising from inadequate information, limited involvement in decision-making and disruptions to routines,^43 44^ which are concepts consistent with the qualitative findings of this review.

Within this review, many concepts were described across multiple studies (online supplemental table S7), suggesting a degree of consistency in reported parental experiences. Moreover, within the quantitative studies on parental well-being, several findings offer empirical support for concepts identified in the qualitative synthesis, with studies finding significant associations between parents’ reported experiences and: (1) (perceived) differences in care practice or competence between units (perceived safety and quality of care), (2) preparing parents for transfer and answering their questions (communication and information access), (3) infant problems post-transfer (clinical rationale and impact) and (4) prior contact with the receiving unit (continuity and familiarity).^35 36 38^

However, with the interpretation of our findings, it should be noted that for the qualitative components, most studies relied on retrospective data collection, introducing the potential for recall bias, as parental experiences were reported after discharge and may have been influenced by time or subsequent experiences, especially in studies that collected data months to years after discharge.^28 30 37^ For the quantitative data, some studies were affected by non-response bias, particularly those with low response rates,^36 38^ which may limit the generalisability of reported parental experiences, as parents who chose to respond may differ from those who did not.

Importantly, this review identifies several modifiable factors that may improve parental experiences. Preparing parents for and involving parents in transport, and familiarising parents with the receiving unit in advance represent key opportunities to improve parental well-being throughout the transport process. Supportive interventions such as skin-to-skin contact, communication strategies and video calls have been reported to benefit parents.^45^ Given that two studies included in this review report that socioeconomic and educational factors may influence parents’ perceptions of transport,^25 36^ a phenomenon also described across other healthcare settings,^46–48^ these interventions should be tailored to the needs of parents of different socioeconomic or cultural backgrounds to ensure equitable support.

### Limitations and recommendations

This review has several limitations. Clinical findings remain conflicting, ranging from no effect to either positive or negative effects of transport. Variability in study designs, composition of study populations, outcome measures and the interval of outcome assessment or follow-up (online supplemental table S4), limits comparability and likely contributed to these conflicting results. In addition, most studies did not adjust their findings for pre-transport clinical state, weight, age, respiratory support or comorbidities. Varying eligibility criteria for interventions like back-transport limit the comparability of results and may partly explain the variability in infant responses to and recovery from neonatal transport. Because this review did not set requirements for study type, size or methodological quality, some included studies had small samples, limiting the use of statistical testing and complicating the interpretation of results.^31 39 40^ Moreover, heterogeneity in outcome reporting prevented meta-analysis or certainty-of-evidence assessment, which means that many of the quantitative findings in this review are effectively single-study observations. Findings regarding qualitative parental well-being outcomes are limited by an underrepresentation of fathers in most study cohorts. In addition, while the research team represents diverse clinical and academic perspectives, the team’s composition may have introduced bias in the synthesis of qualitative outcomes. As no date restrictions were applied during study selection, several included studies were conducted more than two decades ago.^21 25 34 36^ Given changes in neonatal care and transport practices over time, the applicability of these findings to current clinical settings may be limited for both infant health and parental well-being outcomes.

Despite these limitations, this review offers a consolidated overview of the available evidence on the effects of neonatal non-emergency inter-hospital transport. This is a topic of growing relevance as capacity constraints and efforts to centralise specialised neonatal care such as retinopathy of prematurity treatment increase the reliance on such forms of neonatal transport. A remaining research gap is the lack of consensus on the level of clinical stability that permits non-emergency transport without compromising infant health. More research on the determinants of physiological deterioration during non-emergency transport is needed to clarify which infants are likely to tolerate transfer and which may require additional support or a transport delay. Such evidence would support decision-making at referring NICUs and would also inform receiving healthcare providers about expected care needs of these infants on arrival. In addition, neonatal transport services should co-design and implement interventions that reduce parental stress and support their emotional well-being. Communication appears to be one of the most important determinants of parental (dis)satisfaction during their child’s transition and therefore represents an important area for improvement. The widespread availability of mobile phones and digital communication tools offers new ways to improve parent-provider interactions and parental involvement throughout the transport process. For example, digital communication tools could enable real-time communication with care providers or a visual connection to their infant during transport. These forms of telemedicine may also support the communication between referring and receiving units and aid in patient handover. However, despite its growing use in other areas of neonatal care, telemedicine has not yet been applied to inter-hospital neonatal transport at scale.^49^ Future research should therefore examine whether, and in what ways, these technologies could be implemented into practice.

## Conclusion

Within the available evidence, no consistent association between non-emergency inter-hospital transport and adverse long-term clinical outcomes was identified; however, the evidence base is limited, and short-term physiological deterioration during transport has been reported, particularly during prolonged transport. Across studies on parental well-being, stress and anxiety recurred within parent-reported experiences, despite the plannable nature of non-emergency transport. Adequate communication, preparation and reassurance from healthcare providers, as well as active parental involvement in the transfer process, may contribute to more positive parental experiences.

## Supplementary material

10.1136/bmjpo-2026-004846 online supplemental table 1

10.1136/bmjpo-2026-004846 online supplemental table 2

10.1136/bmjpo-2026-004846 online supplemental table 3

10.1136/bmjpo-2026-004846 online supplemental table 4

10.1136/bmjpo-2026-004846 online supplemental table 5

10.1136/bmjpo-2026-004846 online supplemental table 6

10.1136/bmjpo-2026-004846 online supplemental table 7

## Data Availability

All data relevant to the study are included in the article or uploaded as supplementary information.
