# Supplementary material for: Exploring the impact of non-emergency inter-hospital neonatal transport on infants and their parents: a mixed-methods systematic review
Source: BMJ Paediatr Open. 2026 Oct 28;10(1):e004846. doi: 10.1136/bmjpo-2026-004846 (PMC13723885; doi:10.1136/bmjpo-2026-004846)
Supplement: Supplementary file 2 [file bmjpo-10-1-s002.pdf]

Exploring the Impact of Non-Emergency Inter-Hospital Neonatal Transport on Infants and Their Parents: A Mixed-Methods Systematic Review. L.A. Derks, M.L.J. Partouns, M.R. Koolen-De Koninck, A.J. Apperloo, S.A. Obermann-Borst, A.M. Tjiam, H.R. Taal, J.H.L. Wagenaar. Corresponding author: J.H.L. Wagenaar, j.wagenaar@erasmusmc.nl. Department of Neonatal and Paediatric Intensive Care, Division of Neonatology, Erasmus MC, Rotterdam, The Netherlands

Supplemental Table S2: Inter-rater reliability

Title and abstract screening					
**Screening authors***	**Articles screened (n)**	**Agree (n)**	**Disagree (n)**	**Agreement (%)**	**Cohen's Kappa**
JHLW, MRK	74	66	8	89.2	0.28
LAD, AJA	137	63	74	46.0	0.06
LAD, MRK	1103	981	122	88.9	0.41
MRK, MLJP	282	265	17	94.0	0.46
LAD, MLJP	922	809	113	87.7	0.60
JHLW, LAD	67	52	15	77.6	0.44
JHLW, AJA	89	68	21	76.4	0.22
MLJP, AJA	35	14	21	40.0	-0.04
JHLW, MLJP	72	60	12	83.3	0.43
MRK, AJA	30	24	6	80.0	0.13
Total	2811	2402	409	85.5	N/A
**Full text screening**					
**Screening authors***	**Articles screened (n)**	**Agree (n)**	**Disagree (n)**	**Agreement (%)**	**Cohen's Kappa**
LAD, MLJP	228	220	8	96.5	0.48
LAD, MRK	176	161	15	91.5	0.50
LAD, JHLW	7	7	0	100.0	N/A†
MLJP, MRK	20	20	0	100.0	1.00
JHLW, MRK	4	4	0	100.0	N/A†
JHLW, MLJP	3	3	0	100.0	N/A†
Total	438	415	23	94.7	N/A

*Professional roles of screening authors: LAD, PhD-student; MLJP, medical student; MRK, NICU research nurse; AJA, senior NICU nurse; JHLW, medical doctor & PhD-student. †Cohen's Kappa could not be calculated because all articles screened by the screening author pair were unanimously excluded. Abbreviations: N/A, not available; n, number.
