# Supplementary material for: Exploring the impact of non-emergency inter-hospital neonatal transport on infants and their parents: a mixed-methods systematic review
Source: BMJ Paediatr Open. 2026 Oct 28;10(1):e004846. doi: 10.1136/bmjpo-2026-004846 (PMC13723885; doi:10.1136/bmjpo-2026-004846)
Supplement: Supplementary file 3 [file bmjpo-10-1-s003.pdf]

Supplemental Table S3: Infant characteristics

First author (year)	Population (n)	Female sex (%)	GA (weeks)	BW (grams)	PMA transport (weeks)	PNA transport (days)	Weight transport (grams)	Support at transport
Arora (2014)	106	41	26.0 (SD 2)	777 (SD 159)	33 (SD 6)	56 (SD 45)	1610 (SD 924)	NA
Attar (2005)	104	NA	33.0 (IQR 31, 35)	1955 (IQR 1545, 2655)	NA	NA	NA	NA
Chakraborty (2022)	201	50	Inborn: 31.0 (IQR 30, 33)<br>Outborn: 36.0 (IQR 32,37)	Inborn: 1305 (IQR 1155,1485)<br>Outborn: 2000 (IQR 1400,2615)	Inborn: 34.0 (IQR 32, 35)<br>Outborn: 37.0 (IQR 34, 39)	NA	Inborn: 1386 (IQR 1269, 1510)<br>Outborn: 2100 (IQR 1500, 2800)	NA
de Preud'homme d'Hailly de Nieuport (2017)	91	NA	30.4 (SD 2.7)	1446 (SD 492)	NA	19 (SD 21.2)	NA	42% no respiratory support or LFNC<br>58% HFNC or CPAP
Hennequin, SSC (2018)	94	NA	32.0 (IQR 25, 42)	1770 (IQR 680, 3850)	35.0 (IQR 32, 53)	17 (IQR 1, 92)	2220 (IQR 1180, 4775)	No respiratory support, no IV lines
Karlsson (2012)	16	NA	33.0 (SD 3.9)	NA	NA	17 (SD 25)	2507 (SD 1289)	38% assisted ventilation<br>13% nCPAP<br>25% supplemental oxygen<br>25% room air
Lainwala (2007)	504	51	26.6 (SD 2.1, R 22–35)	809 (SD 140, R 411–1000)	34.7 (SD 4.5, R 24.9–69.7)	56.8 (SD 35.8, R 1–327)	1600 (SD 700, R 600–7200)	NA
Lynch (1988)	55	49	32 (SD N/A)	1614 (SD N/A)	NA	18.5 (SD 15.3)	1700 (SD 455)	35% IV fluids<br>25% supplemental oxygen, no CPAP or assisted ventilation
van den Berg, SSC (2022)	16	62.5	34.3 (R 24.7–41.0)	2240 (R 450–3450)	36.6 (R 32.9–41.9)	NA	2456 (R 1262–3574)	25% supplemental oxygen 0.25-0.5 L/min (n=4)
van den Berg (2022)	8	25	34.4 (R 30.9–41.0)	2682 (R 1990–3585)	35.9 (R 31.7-42.7)	NA	2601 (R 1770–4020)	12.5% supplemental oxygen 0.4 L/min (n=1)
Zwissig (2019)	20	60	<32: 8 (40%)<br>32-33: 4 (20%)<br>34-36: 5 (25%)<br>≥37: 3 (15%)	NA	32-33: 5 (25%)<br>34-36: 8 (40%)<br>≥37: 7 (35%)	NA	NA	10% HFNC (n=2)<br>10% nCPAP (n=2)
Chant (2024)	20	NA	R 24.0–35.9	NA	R 31.3–44.4	R 4–141	NA	NA
De Bijl-Marcus (2025)	46	NA	NA	NA	NA	NA	NA	NA
Dodds-Azzopardi (1995)	24	NA	NA	NA	NA	NA	NA	NA
Donohue (2009)	133	47	28.3 (95%CI 27.8–28.7)	1060 (95%CI 1014–1104)	34.25 (IQR 32.6, 37.5)	40 (IQR 21, 73)	1770 (IQR 1445, 2290)	52% supplemental oxygen
Dosani (2021)	140	NA	NA	NA	NA	NA	NA	NA
Granrud (2014)	11	NA	28.0 (R 26–32)	NA	NA	NA	NA	NA
Hanrahan (2007)	12	NA	NA	NA	NA	NA	NA	NA
Helder (2012)	24	66.7	30.7 (IQR 26.0, 39.4)	1780 (IQR 635, 4000)	NA	NA	NA	Transport criteria: > 1000 grams, CPAP or less respiratory support
Kuhnly (1993)	9	NA	NA	NA	NA	NA	NA	NA
Murthy (2022)	140	NA	NA	NA	NA	NA	NA	NA
Slattery (1998)	143	41	81% preterm	1900 (R 503–4990), 2070 (SD 950)	NA	NA	NA	NA
Sommer (2015)	6	NA	R 23.9–29.0	NA	NA	R 21–84	NA	NA
van den Berg (2011)	223	NA	NA	NA	NA	NA	NA	NA

Mean (SD and/or R or 95%CI), Median (IQR: 25th quartile, 75th quartile), R, or %. Population includes the number of transported infants, but does not include (if applicable) non-transported controls. Abbreviations: BW, birthweight; CI, confidence interval; (n)CPAP, (nasal) continuous positive airway pressure; GA, gestational age; IQR, interquartile range; IV, intravenous; HFNC, high flow nasal cannula; LFNC, low flow nasal cannula; NA, not applicable; PMA, postmenstrual age; PNA, postnatal age; R, range; SD, standard deviation; SSC; skin-to-skin contact.
