# Supplementary material for: Exploring the impact of non-emergency inter-hospital neonatal transport on infants and their parents: a mixed-methods systematic review
Source: BMJ Paediatr Open. 2026 Oct 28;10(1):e004846. doi: 10.1136/bmjpo-2026-004846 (PMC13723885; doi:10.1136/bmjpo-2026-004846)
Supplement: Supplementary file 4 [file bmjpo-10-1-s004.pdf]

Supplemental Table S4: Infant health outcome measures

Category	Outcome	First author (year)
**Pre- vs. post-transport state**	1. Transport Risk Index of Physiologic Stability score (TRIPS; temperature, respiratory status, systolic blood pressure, and response to stimuli) measured upon departure and on arrival	Arora (2014)
	2. Respiratory rate measured upon departure and on arrival	Hennequin (2018)
	3. Respiratory rate measured upon departure, on arrival and 48 hours after transport	Van den Berg (2022)
	4. Respiratory rate measured at six time points between departure and arrival	Zwissig (2019)
	5. Respiratory support type 1 day before and 7 days after transport	de Preud'homme d'Hailly de Nieuport (2017)
	6. Oxygen requirement 1 day before and 7 days after transport	de Preud'homme d'Hailly de Nieuport (2017)
	7. Oxygen saturation measured upon departure and on arrival	Hennequin (2018)
	8. Oxygen saturation measured upon departure, on arrival and 48 hours after transport	Van den Berg (2022)
	9. Oxygen saturation measured at six time points between departure and arrival	Zwissig (2019)
	10. Heart rate measured upon departure and on arrival	Hennequin (2018)
	11. Heart rate measured upon departure, on arrival and 48 hours after transport	Van den Berg (2022)
	12. Heart rate measured at six time points between departure and arrival	Zwissig (2019)
	13. Heart rate measured continuously throughout transport process	Karlsson (2012)
	14. Heart rate variability measured continuously throughout transport process	Karlsson (2012)
	15. Number of bradycardia episodes 1 day before and 7 days after transport	de Preud'homme d'Hailly de Nieuport (2017)
	16. Body temperature 1 day before and 7 days after transport	de Preud'homme d'Hailly de Nieuport (2017)
	17. Body temperature measured upon departure and on arrival	Hennequin (2018)
	18. Body temperature measured upon departure, on arrival and 48 hours after transport	Van den Berg (2022)
	19. Astrid Lindgren Children’s Hospital Pain Scale (ALPS) measured upon departure, on arrival and 48 hours after transport	Van den Berg (2022)
	20. Premature Infants Pain Profile-Revised (PIPP-R) measured at six time points between departure and arrival	Zwissig (2019)
	21. Comfort Behaviour (CB) measured at six time points between departure and arrival	Zwissig (2019)
	22. Salivary cortisol levels measured upon departure and on arrival	Zwissig (2019)
**Transported vs. non-transported infants**	1. Number of apnoea episodes during convalescence* until discharge home	Lynch (1988)
	2. Number of bradycardia episodes during convalescence until discharge home	Lynch (1988)
	3. Oxygen requirement during convalescence until discharge home	Lynch (1988)
	4. Feeding intolerance during convalescence until discharge home	Lynch (1988)
	5. Daily weight gain during convalescence until discharge home	Lynch (1988)
	6. New clinical problems† during convalescence until discharge home	Lynch (1988)
	7. Return to higher-level unit during convalescence until discharge home	Lynch (1988)
	8. Mortality at 3 months corrected age	Chakraborty (2022)
	9. Mortality at 6 months corrected age	Lainwala (2007)
	10. Mortality at 18-22 months corrected age	Lainwala (2007)
	11. Total length of hospital stay (LOS)	Attar (2005)
	12. Total length of hospital stay (LOS)	Lainwala (2007)
	13. Number of readmissions within 2 weeks after discharge	Attar (2005)
	14. Number of readmissions until 3 months corrected age	Chakraborty (2022)
	15. Number of readmissions until 18-22 months corrected age	Lainwala (2007)
	16. Number of readmissions until 12 months post-natal age	Lainwala (2007)
	17. Number of emergency room visits until 3 months corrected age	Chakraborty (2022)
	18. Number of outpatient visits until 3 months corrected age	Chakraborty (2022)
	19. Weight at 18-22 months corrected age	Lainwala (2007)
	20. Height at 18-22 months corrected age	Lainwala (2007)
	21. Head circumference at 18-22 months corrected age	Lainwala (2007)
	22. Mental developmental index (MDI) at 18-22 months corrected age	Lainwala (2007)
	23. Psychomotor developmental index (PDI) at 18-22 months corrected age	Lainwala (2007)
	24. Neurodevelopmental impairment (NDI; MDI < 70, PDI < 70, cerebral palsy, hearing impairment, or blindness) at 18-22 months corrected age	Lainwala (2007)

*Convalescence was defined as the time admitted to the receiving hospital after back-transport. For non-transported controls, “convalescence” commenced when infants met the criteria for back-transport. Criteria for back-transport were: 1) Acute clinical problems resolved; 2) Extubated for a minimum of 48 hours; 3) Fi02 0.40 or scant oxygen by nasal cannula; 4) Caloric intake (enteral and parenteral) 40 kcal/kg/d; 5) Absence of feeding intolerance (i.e., emesis, abdominal distention, and gastric residuals 3 mL/kg) for preceding 96 hours; 6) Absence of episodes of apnoea and bradycardia requiring bag and mask resuscitation for preceding 96 hours; 7) Temperature stable in controlled environment or bassinet; 8) If phototherapy required, stable or decreasing indirect bilirubin level for 48 hours; 9) No arterial or central venous catheters in place. †Study includes minor problems (mild apnoea and bradycardia, constipation, hyperbilirubinemia, oral or perianal monilial infections, suspected sepsis, conjunctivitis, diaper rash, and instances of vomiting, diarrhea, or occult blood in the stools) and major problems (aspiration pneumonia, respiratory arrest, seizures, hyperalimentation, suspected necrotizing enterocolitis, gastroesophageal reflux, sepsis, hypertension, and suspected incarcerated inguinal hernia).
