# Supplementary material for: Exploring the impact of non-emergency inter-hospital neonatal transport on infants and their parents: a mixed-methods systematic review
Source: BMJ Paediatr Open. 2026 Oct 28;10(1):e004846. doi: 10.1136/bmjpo-2026-004846 (PMC13723885; doi:10.1136/bmjpo-2026-004846)
Supplement: Supplementary file 5 [file bmjpo-10-1-s005.pdf]

Exploring the Impact of Non-Emergency Inter-Hospital Neonatal Transport on Infants and Their Parents: A Mixed-Methods Systematic Review. L.A. Derks, M.L.J. Partouns, M.R. Koolen-De Koninck, A.J. Apperloo, S.A. Obermann-Borst, A.M. Tjiam, H.R. Taal, J.H.L. Wagenaar. Corresponding author: J.H.L. Wagenaar, j.wagenaar@erasmusmc.nl. Department of Neonatal and Paediatric Intensive Care, Division of Neonatology, Erasmus MC, Rotterdam, The Netherlands

Supplemental Table S5: Parent characteristics

First author (year)	Population (n)	Mothers (%)	Parental age, mean (SD or 95%CI)	Primiparae (%)	Married (%)	White ethnicity (%)	Educational attainment	Socioeconomic status or income
Chant (2024)	24	Mother: 65.0<br>Mother + father: 20.0<br>Father: 15.0	NA	NA	NA	NA	NA	NA
De Bijl-Marcus (2025)	39	NA	NA	NA	NA	NA	NA	NA
Dodds-Azzopardi (1995)	44	54.5	Mothers: 30.8 (SD 4.8)<br>Fathers: 31.8 (SD 5.9)	59.1	86.4	NA	NA	NA
Donohue (2009)	133	97.0	Parents: 27 (95CI 25-28)	NA	49.0	38.0	Less than high school: 21%<br>High school: 37%<br>Some college: 19%<br>College degree: 22%	70% private insurance
Dosani (2021)	140	91.2	NA	NA	94.9	69.6	High school and College: 31.1%,<br>Undergraduate, Masters, PhD: 68.9%	< 20,000 to < 60,000 CAD: 25 (19.1%)<br>≥ 61,000 CAD: 106 (80.9%)
Granrud (2014)	20	55.0	NA	NA	NA	NA	NA	NA
Hanrahan (2007)	12	83.3	NA	NA	NA	83.3	NA	All parents were Medicaid recipients
Helder (2012)	28	Mother: 66.6<br>Mother + father: 33.3	Mothers: 31.2 (SD 4.6)	NA	100	75.0	NA	NA
Kuhnly (1993)	14	Mother + father: 100.0	Mothers: 28.3 (SD 3.2)<br>Fathers: 30.6 (SD 2.7)	57.1	100	NA	NA	NA
Murthy (2022)	140	91.2	NA	NA	94.9	69.6	High school and college: 31.1%<br>Undergraduate, Masters, PhD: 68.9%	< 20,000 to < 60,000 CAD: 25 (19.1%)<br>≥ 61,000 CAD: 106 (80.9%)
Slattery (1998)	143	100	Mothers: 28 (SD 6.0)<br>Fathers: 31 (SD 6.5)	42.0	69.9	97.0	NA	No income: 3.5%,<br><20,000 USD: 28.0%.<br>20,001-40,000 USD: 27.3%,<br>40,001-60,000 USD: 19.6%,<br>Unknown: 7.7%
Sommer (2015)	6	83.3	NA	NA	NA	NA	NA	NA
van den Berg (2011)	223	NA	NA	NA	NA	NA	NA	NA

Population includes the number of individual parents. Abbreviations: CAD, Canadian dollars; 95%CI, 95% confidence interval; SD, standard deviation; USD, United States dollars.
