# Supplementary material for: Exploring the impact of non-emergency inter-hospital neonatal transport on infants and their parents: a mixed-methods systematic review
Source: BMJ Paediatr Open. 2026 Oct 28;10(1):e004846. doi: 10.1136/bmjpo-2026-004846 (PMC13723885; doi:10.1136/bmjpo-2026-004846)
Supplement: Supplementary file 6 [file bmjpo-10-1-s006.pdf]

Exploring the Impact of Non-Emergency Inter-Hospital Neonatal Transport on Infants and Their Parents: A Mixed-Methods Systematic Review. L.A. Derks, M.L.J. Partouns, M.R. Koolen-De Koninck, A.J. Apperloo, S.A. Obermann-Borst, A.M. Tjiam, H.R. Taal, J.H.L. Wagenaar. Corresponding author: J.H.L. Wagenaar, j.wagenaar@erasmusmc.nl. Department of Neonatal and Paediatric Intensive Care, Division of Neonatology, Erasmus MC, Rotterdam, The Netherlands

Supplemental Table S6: Qualitative parental wellbeing outcomes

	Parental satisfaction with or approval of transport	Parental dissatisfaction with or opposition to transport	Parental satisfaction with convalescent care after transfer	Parental dissatisfaction with convalescent care after transfer
**Categories**	**Concepts**	**Concepts**	**Concepts**	**Concepts**
Parental or infant characteristics	➢ Receiving centre closer to home (Donohue, Hanrahan, Chant)	➢ Receiving centre is farther from home (Donohue, Kuhnly)<br>➢ Receiving centre is less accessible with public transport (Donohue)<br>➢ Infant shows (temporary) clinical deterioration after transport (Helder, Granrud, Kuhnly)	➢ Receiving centre is closer to home, especially if parents have other children as well (Granrud)	➢ Receiving centre is farther from home (Donohue)
Parental perceptions	➢ The reason for transport is clinical improvement of the infant (Donohue, Helder, Dosani, Chant, Kuhnly)<br>➢ Transfer means the infant will get more attention (Donohue, Chant)<br>➢ Transfer creates room for another preterm or sick infant (Donohue, Chant, Kuhnly)<br>➢ Feeling of being “partners in crime” with staff at the transferring centre (Helder)<br>➢ Satisfied with the quality of care at the transferring unit during preparation for transport (Helder)<br>➢ Transferring unit is well organized during preparation for transport (Helder)	➢ Preparation for transport occurs in a hectic and busy transferring unit (Helder)<br>➢ Transport is not necessary (Helder, Kuhnly)<br>➢ Transport is a source of fear:<br>○ Fear of the unknown (Donohue, Helder, Bijl, Kuhnly)<br>○ Fear of the infant not being ready for transport (Donohue, Helder, Granrud)<br>○ Fear of transport being harmful to the infant (Helder, Granrud, Bijl, Chant)<br>○ Fear of accidents (Donohue, Granrud, Kuhnly)<br>○ Fear that the receiving unit does not have the facilities to meet the child’s needs (Chant, Kuhnly)<br>➢ Transport is seen as a loss of:<br>○ Established routines at the transferring unit (Helder, Sommer, Chant)<br>○ Attachment to the transferring unit, its staff and other parents (Hanrahan, Donohue, Sommer, Chant, Kuhnly)<br>○ Feelings of control (Granrud, Sommer)<br>➢ Transport causes feelings of separation from the infant (Granrud, Chant)	➢ The receiving unit is more pleasant than the transferring unit:<br>○ Care is less intensive (Donohue)<br>○ Environment is quieter and calmer (Donohue, Helder)<br>○ There are more windows (Kuhnly)<br>○ Parents have to relate to less people (Donohue, Granrud)<br>○ Parents receive more attention and communication (Donohue)<br>➢ Feeling a more intense awareness of having a baby (Granrud)<br>➢ Satisfaction with the quality of care and care professionals’ understanding of the infant’s needs (Murthy, Helder, De Bijl, Kuhnly)	➢ The receiving unit is less pleasant than the transferring unit:<br>○ Poorer communication (Donohue)<br>○ Poorer (perceived) quality of nursing care (Donohue, Granrud)<br>○ Dissatisfaction with reduced involvement in the care of their child (Hanrahan, Kuhnly)<br>○ Dissatisfaction with reduced nurse-to-patient ratio, response time or level of personal attention (Hanrahan, Helder, Kuhnly)<br>○ Dissatisfaction with changes to (feeding) routines (Hanrahan, Helder, Kuhnly)<br>➢ Feeling less safe at the receiving centre due to less strict infection control and visitation policies (Sommer)<br>➢ Feeling less like parents due to loss of parental responsibilities (Sommer)<br>➢ Negative encounters with staff post-transfer (Dosani)
Provider behaviour	➢ Provides written information about the receiving centre (Chant)<br>➢ Facilitates a visit to the receiving centre for parents (Helder, Chant)<br>➢ Facilitates personal contact between parents and staff at the receiving centre (Bijl, Chant)<br>➢ Facilitates personal contact between parents and ambulance personnel (Bijl)<br>➢ Facilitates for parents to accompany the child during transport (Helder, Bijl, Chant)<br>➢ Actively asks parents about their concerns and involves parents in decision-making (Bijl, Chant)<br>➢ Facilitates that a familiar care professional accompanies the child during transport (Bijl)<br>➢ Prepares parents for uncertainties in transport process and explains the reasons behind them (Bijl)<br>➢ Provides a timely notice (e.g. 24h) before transport (Dosani, Chant)<br>➢ Reassures parents transfer is a step in the right direction (Dosani, Chant)<br>➢ Informs parents when the child has arrived (Granrud)	➢ Fails to provide accurate, adequate and timely communication about:<br>○ The actual transfer process, transfer destination and other transfer logistics (Helder, Dosani, Dodds, Chant)<br>○ The possible clinical implications of transport (Dosani, Granrud)<br>○ What to expect at the receiving unit (Granrud, Dosani, Hanrahan, Kuhnly)<br>○ The timing of transport (Dodds, Sommer, Bijl, Hanrahan, Helder, Dosani, Chant, Kuhnly)<br>➢ Provides contradictory information about the criteria for transport and the level of care that can be delivered at the receiving unit (Granrud, Bijl)<br>➢ Infant is accompanied by an unfamiliar nurse during transport (Granrud)	➢ Provide positive and personal communication, including compassion, respect and acknowledgement (Hanrahan, Granrud, Kuhnly)<br>➢ Provide family centered care (Hanrahan)<br>➢ Friendly reception of parents (Helder)<br>➢ Allow more involvement of parents in everyday care (Helder, Granrud, Donohue)<br>➢ Adequate preparation for discharge home (Helder)<br>➢ Regains the trust of the parents (Helder, Chant)	➢ Fails to provide continuity of care (Dodds)<br>➢ Fails to prepare parents for the different level of care (Hanrahan, Helder, Dodds)<br>➢ Denies parents participation in care that they were previously allowed (Hanrahan, Sommer)<br>➢ Being poorly informed about the condition of the child (Helder)
