# Supplementary material for: Exploring the impact of non-emergency inter-hospital neonatal transport on infants and their parents: a mixed-methods systematic review
Source: BMJ Paediatr Open. 2026 Oct 28;10(1):e004846. doi: 10.1136/bmjpo-2026-004846 (PMC13723885; doi:10.1136/bmjpo-2026-004846)
Supplement: Supplementary file 7 [file bmjpo-10-1-s007.pdf]

Exploring the Impact of Non-Emergency Inter-Hospital Neonatal Transport on Infants and Their Parents: A Mixed-Methods Systematic Review. L.A. Derks, M.L.J. Partouns, M.R. Koolen-De Koninck, A.J. Apperloo, S.A. Obermann-Borst, A.M. Tjiam, H.R. Taal, J.H.L. Wagenaar. Corresponding author: J.H.L. Wagenaar, j.wagenaar@erasmusmc.nl . Department of Neonatal and Paediatric Intensive Care, Division of Neonatology, Erasmus MC, Rotterdam, The Netherlands

Supplemental Table S7: Qualitative synthesis

Author (year)[ref]	Geographical accessibility	Clinical rationale and impact	Communication and information access	Continuity and familiarity	Fear and psychological stress	Routines and relationships	Parental involvement, agency, and control	Environment and unit atmosphere	Perceived safety and quality of care
**Kuhnly (1993)[42]**	✓	✓	✓		✓	✓	✓	✓	
**Dodds-Azzopardi (1995)[25]**			✓	✓					
**Hanrahan (2007)[29]**	✓		✓			✓	✓		✓
**Donohue (2009)[26]**	✓	✓	✓		✓	✓	✓	✓	✓
**Helder (2012)[30]**		✓	✓	✓	✓	✓	✓	✓	✓
**Granrud (2014)[28]**	✓	✓	✓	✓	✓	✓	✓	✓	✓
**Sommer (2015)[37]**			✓			✓	✓		✓
**Dosani (2021)[27]**		✓	✓	✓		✓			
**Chant (2024)[41]**	✓	✓	✓	✓	✓	✓	✓		✓
**De Bijl-Marcus (2025)[23]**			✓	✓	✓		✓		✓

Key concepts*

a. **Geographical accessibility:** Location of the receiving unit in relation to the family's home and availability of public transport.
b. **Clinical rationale and impact:** Medical reasons for transfer and its effects on the infant's clinical condition.
c. **Communication and information access:** Clarity, timing, and consistency of information shared with parents before, during and after transport, and during convalescent care.
d. **Continuity and familiarity:** Consistency of care practices and presence of familiar care providers at transferring and receiving units, and during transfer.
e. **Fear and psychological stress:** Parental fears related to the (unfamiliar) transfer process and the care at the receiving unit.
f. **Routines and relationships:** Changes to established care routines and separation from familiar care providers or peers at the receiving unit compared to the transferring unit.
g. **Parental involvement, agency, and control:** Opportunities for parents to participate in care, take a "parental role" and influence decisions during preparation, transport, and convalescent care.
h. **Environment and unit atmosphere:** Sensory and social aspects of the care setting, including noise, business and intensity of care at the receiving unit compared to the transferring unit.
i. **Perceived safety and quality of care:** Parental perceptions of hospital policies and competence of care providers at the receiving unit compared to the transferring unit.

Interpretations

a. When parents transition to a new unit, they lose the comfort of a place where they felt secure, understood their role in everyday care, and had strong connections with care providers and other families, which contributes to feelings of stress and anxiety.
b. The transition to another unit can be psychologically taxing for parents, who may feel fearful, uncertain, powerless, and separated from their child, which leads to heightened feelings of distress.
c. Parental perceptions of convalescent care are shaped by comparisons with their child's previous care setting and can be positively influenced by adequate preparation for the change in level of care and hospital policies.
d. Parental experiences during transport and convalescent care can be significantly enhanced through 1) clear, timely, and accurate communication, 2) prior introduction to and familiarisation with the receiving unit, 3) reassurance throughout the transfer process, 4) active parental involvement in the planning and execution of transfer and subsequent convalescent care 5) maintaining or improving the accessibility of the child, and 6) early restoration of trust and parents' role in care.

Final line-of-argument

Parents often experience stress and anxiety throughout the transport process, regardless of the level of clinical stability of the infant. Parental wellbeing during the transition from one unit to another is influenced by psychological factors, environmental factors, and provider behaviour. Managing parental stress during this transition requires clear and timely communication, adequate preparation, and reassurance of infant safety. Parents also benefit from opportunities to maintain an active role in the entire process and from efforts to reestablish relationships, routines, and trust at the new care setting.

*Key concepts are derived from second order interpretations from the original studies presented in Supplemental Table S6.
