# Supplementary material for: Exploring the impact of non-emergency inter-hospital neonatal transport on infants and their parents: a mixed-methods systematic review
Source: BMJ Paediatr Open. 2026 Oct 28;10(1):e004846. doi: 10.1136/bmjpo-2026-004846 (PMC13723885; doi:10.1136/bmjpo-2026-004846)
Supplement: Supplementary file 8 [file bmjpo-2026-004846.reviewer_comments.pdf]

PEER REVIEW HISTORY

BMJ Paediatrics Open publishes all reviews undertaken for accepted manuscripts. Reviewers are asked to complete a checklist review form and are provided with free text boxes to elaborate on their assessment. These free text comments are reproduced below.

ARTICLE DETAILS

**TITLE (PROVISIONAL)**	Exploring the Impact of Non-Emergency Inter-Hospital Neonatal Transport on Infants and Their Parents: A Mixed-Methods Systematic Review
**AUTHORS**	Derks, Lizanne A.<br>Partouns, Myrthe L.J.<br>Koolen-De Koninck, Margreet R.<br>Apperloo, Anna J.<br>Obermann-Borst, Sylvia A.<br>Tjiam, Angela M.<br>Taal, H. Rob<br>Wagenaar, Josephine H.L.

VERSION 1 – REVIEW

**REVIEWER NAME**	Liu, Shiyao
**REVIEWER AFFILIATION**	University of Lancashire, School of Medicine
**REVIEWER CONFLICT OF INTEREST**	No Conflict of Interest
**AI DISCLOSURE**	No, I have not used AI
**DATE REVIEW RETURNED**	04-Jun-2026

**GENERAL COMMENTS**	The authors have undertaken a comprehensive and detailed review of the topic of neonatal transport. The review addresses an important clinical question and provides valuable insights into the current evidence base. I have a few suggestions that may further improve the clarity and readability of the manuscript.<br><br>1. The Introduction currently has relatively few citations. Factual statements that are not common knowledge or original interpretations by the authors would benefit from appropriate referencing.<br><br>2. The Results section would benefit from a clearer synthesis of the evidence. At present, parts of the narrative read somewhat like a study-by-study description of findings. It may be helpful to begin each subtheme with a brief summary statement describing the overall direction of the evidence.<br><br>In addition, grouping findings by outcome rather than reporting studies sequentially may help readers identify patterns across studies more easily.<br><br>For example, in Section 3.3.4, the opening statement suggests that transported and non-transported infants did not differ significantly. However, this is immediately followed by findings favouring transport. As a reader, it becomes difficult to reconcile the overall conclusion with the individual study findings.

	3. Some sections of the Results appear to include interpretation of findings. It may be preferable to focus on reporting the findings themselves in the Results section and reserve interpretation and discussion of possible mechanisms for the Discussion section.<br><br>4. Some conclusions and interpretations in the Discussion appear stronger than the underlying evidence may support. While the overall synthesis allows broad conclusions to be drawn, the relatively small number of studies, heterogeneity in findings, and variation in definitions and outcome measures may affect the certainty of the evidence. The authors may wish to consider using more cautious language when interpreting the findings and acknowledging the uncertainty associated with the available evidence.

**REVIEWER NAME**	Haines, Charlotte
**REVIEWER AFFILIATION**	University Of Lancashire
**REVIEWER CONFLICT OF INTEREST**	No Conflict of Interest
**AI DISCLOSURE**	No, I have not used AI
**DATE REVIEW RETURNED**	10-Jun-2026

**GENERAL COMMENTS**	Thank you for the opportunity to review this manuscript. This mixed-methods systematic review addresses an increasingly important and clinically relevant topic.<br>(1) There are some minor mis-spellings and formatting errors.<br>(2) Criteria for considering the quality for the MMAT could be presented and consideration of results in the discussion, did this affect confidence in the results etc? The MMAT assessment notes issues with confounding and small heterogeneous samples. I think the discussion could benefit from a more more explicit discussion of confounding, variation in transport indications, changing neonatal transport practices over time etc and consequently what are implications for external validity.<br>(3) I also think it would benefit from greater detail of the qualitative synthesis to improve transparency and reproducibility. For example, clarification of how data was extracted from qualitative and mixed-methods studies for synthesis, whether coding was conducted inductively, deductively? How first-order concepts were generated and translated across studies, how second- and third-order themes were developed and refined, whether coding and interpretation were undertaken independently by more than one reviewer, how disagreements or alternative interpretations were resolved and whether any procedures were implemented to enhance reflexivity or minimise interpretive bias.

VERSION 1 – AUTHOR RESPONSE

Reviewer 1:
Ms. Shiyao Liu, University of Lancashire

Comments to the Author
The authors have undertaken a comprehensive and detailed review of the topic of neonatal transport. The review addresses an important clinical question and provides valuable insights into the current evidence base. I have a few suggestions that may further improve the clarity and readability of the manuscript.

1. The Introduction currently has relatively few citations. Factual statements that are not common knowledge or original interpretations by the authors would benefit from appropriate referencing.

We agree and have revised the Introduction by adding additional references to support factual statements. The relatively low number of references in the original submission was partly due to the journal's limits on word count and references. However, we recognise the importance of adequately supporting factual statements throughout the manuscript and have therefore added the necessary citations. As these additions contributed to exceeding the original reference limit, we have requested permission to exceed the journal's recommended limit.

2. The Results section would benefit from a clearer synthesis of the evidence. At present, parts of the narrative read somewhat like a study-by-study description of findings. It may be helpful to begin each subtheme with a brief summary statement describing the overall direction of the evidence. In addition, grouping findings by outcome rather than reporting studies sequentially may help readers identify patterns across studies more easily. For example, in Section 3.3.4, the opening statement suggests that transported and non-transported infants did not differ significantly. However, this is immediately followed by findings favouring transport. As a reader, it becomes difficult to reconcile the overall conclusion with the individual study findings.

The reviewer has made a very good suggestion to improve the clarity of the synthesis of the evidence and readability of our manuscript. Specifically, each subsection now begins with a brief overarching summary statement describing the direction of the evidence, and findings are now grouped by outcome rather than presented in a study-by-study manner.

3. Some sections of the Results appear to include interpretation of findings. It may be preferable to focus on reporting the findings themselves in the Results section and reserve interpretation and discussion of possible mechanisms for the Discussion section.

We have carefully reviewed the Results section and removed interpretative language. Explanatory or causal statements have been either rephrased in a descriptive manner or moved to the Discussion. The Results now focus exclusively on reporting study findings and synthesis outputs.

4. Some conclusions and interpretations in the Discussion appear stronger than the underlying evidence may support. While the overall synthesis allows broad conclusions to be drawn, the relatively small number of studies, heterogeneity in findings, and variation in definitions and outcome measures may affect the certainty of the evidence. The authors may wish to consider using more cautious language when interpreting the findings and acknowledging the uncertainty associated with the available evidence.

We have revised the Discussion to use more cautious language and to better reflect the limitations and uncertainty of the evidence. Specifically, uncertainty is now explicitly acknowledged, particularly regarding the quantitative outcomes and conclusions have been revised to align more closely with the underlying data (see also response to comment 2 by reviewer 2).

Reviewer 2:
Dr. Charlotte Haines, University Of Lancashire

Comments to the Author
Thank you for the opportunity to review this manuscript. This mixed-methods systematic review addresses an increasingly important and clinically relevant topic.

(1) There are some minor mis-spellings and formatting errors.

We have carefully proofread the manuscript and corrected spelling, grammar, and formatting issues throughout.

(2) Criteria for considering the quality for the MMAT could be presented and consideration of results in the discussion, did this affect confidence in the results etc? The MMAT assessment notes issues with confounding and small heterogeneous samples. I think the discussion could benefit from a more more explicit discussion of confounding, variation in transport indications, changing neonatal transport practices over time etc and consequently what are implications for external validity.

We recognize the MMAT results were insufficiently linked to the described study findings. We have incorporated MMAT findings directly into the “Infant health” and “Parental wellbeing” sections of the Discussion to contextualise the results. The Discussion now explicitly integrates the main methodological limitations identified (e.g., confounding, small sample sizes, heterogeneity) and links them to confidence in the results.

(3) I also think it would benefit from greater detail of the qualitative synthesis to improve transparency and reproducibility. For example, clarification of how data was extracted from qualitative and mixed-methods studies for synthesis, whether coding was conducted inductively, deductively? How first-order concepts were generated and translated across studies, how second- and third-order themes were developed and refined, whether coding and interpretation were undertaken independently by more than one reviewer, how disagreements or alternative interpretations were resolved and whether any procedures were implemented to enhance reflexivity or minimise interpretive bias.

We have expanded the Methods section to improve transparency of the qualitative synthesis: 1) we have added the clarification that an inductive approach was used, 2) we have added detail on how first- and second-order constructs were extracted and translated, 3) we have added a description of the development of higher-order interpretations and the line-of-argument synthesis, 4) we have further specified the role of multiple reviewers in analysis and how disagreements were resolved, 5) we have added an explicit statement addressing reflexivity and minimisation of interpretive bias. In the Results section, we clarified the distinction between first-order (participant data), second-order (author interpretations), and third-order (reviewer synthesis) constructs.

VERSION 2 – REVIEW

**REVIEWER NAME**	Liu, Shiyao
**REVIEWER AFFILIATION**	University of Lancashire, School of Medicine
**REVIEWER CONFLICT OF INTEREST**	No Conflict of Interest
**AI DISCLOSURE**	No, I have not used AI
**DATE REVIEW RETURNED**	06-Aug-2026

**GENERAL COMMENTS**	Thank you to the authors for carefully addressing the comments raised in the previous round of review. I have a small number of remaining comments, mainly concerning the presentation of single-study findings and ensuring that the interpretations and conclusions remain proportionate to the limitations of the available evidence.<br><br>1. I appreciate that the authors generally use cautious language to avoid implying stronger causal relationships than the evidence supports. However, I suggest revising some statements in the Results, particularly where a finding is derived from a single study. Wording such as “One study reported…” may help clarify the source

	of the finding and prevent it from being interpreted as an overall conclusion of the review. For example, in Section 3.3.4, it may be clearer to write: “One study reported lower mortality and readmission rates… After adjustment, the difference…” This makes it explicit that these are findings reported by an individual study rather than interpretations drawn from the evidence.<br><br>I am not suggesting that this wording should be applied throughout the Results. In several places, the authors appropriately synthesise findings across studies. However, where the evidence remains uncertain, slightly more cautious wording could be considered. For example, in Section 3.3.3, “Evidence of possible deterioration included an increased TRIPS score (54% of transported infants) (ref), …” may be more appropriately cautious.<br>2. In several places in the Discussion, findings from a single study appear to be presented as conclusions applying to the overall evidence base. For example, on page 17, lines 377–378, the authors state that “higher hospital readmission rates after discharge home were reported for transported infants compared to non-transported controls.” The subsequent discussion indicates that this finding was primarily derived from Lainwala et al., which included only extremely-low-birthweight infants. When presented in the opening summary without this context, readers may interpret higher readmission as a consistent finding across the review.<br>3. Page 18, line 401: a duplicated comma in this sentence.<br>4. On page 18, lines 418–419, the authors state: “These findings may indicate that any transport-related instability is transient and its effects are limited to the immediate post-transport period.” I am uncertain whether this inference is supported by the available evidence. The absence of statistically significant differences in longer-term studies may reflect insufficient sample sizes, baseline differences between transported and non-transported infants, inadequate adjustment for confounding, or limited statistical precision. It therefore does not necessarily demonstrate that any instability is transient or restricted to the immediate post-transport period. I suggest that the authors reconsider or qualify this interpretation.<br>5. On page 19, line 440, the authors state that “parental distress emerged as a consistent and well-supported finding.” I suggest reconsidering whether “well-supported” appropriately reflects the strength of the evidence, as it may imply a high level of confidence. The number of studies was relatively limited, assessment timepoints varied substantially, and participants were predominantly white, married, college-educated mothers, which limits the applicability of the findings. Recurrence of stress or distress across studies does not establish its magnitude or demonstrate that transport itself caused the distress, particularly in the absence of an appropriate comparator.<br>6. The comments above provide several examples rather than an exhaustive list. I encourage the authors to review the Discussion more broadly to ensure that causal interpretations and overall conclusions are proportionate to the observational, heterogeneous, and frequently single-study evidence.<br>7. The Abstract states that “this review is limited by substantial heterogeneity in outcome reporting, resulting in reliance on mostly unadjusted, single-study findings” Heterogeneity in outcome reporting did not itself result in the included evidence being predominantly unadjusted or derived from single studies. These are separate, although related, limitations. I suggest presenting them in parallel, something like: “The evidence was limited by substantial

	heterogeneity in outcome reporting, predominantly unadjusted analyses, and findings that were frequently derived from single studies.”

**REVIEWER NAME**	Haines, Charlotte
**REVIEWER AFFILIATION**	University Of Lancashire
**REVIEWER CONFLICT OF INTEREST**	No Conflict of Interest
**AI DISCLOSURE**	No, I have not used AI
**DATE REVIEW RETURNED**	27-Jul-2026

**GENERAL COMMENTS**	The authors have addressed each reviewer comment.

VERSION 2 – AUTHOR RESPONSE

Comments to the Author
Thank you to the authors for carefully addressing the comments raised in the previous round of review. I have a small number of remaining comments, mainly concerning the presentation of single-study findings and ensuring that the interpretations and conclusions remain proportionate to the limitations of the available evidence.

We want to thank the reviewer for the careful review of our revised manuscript and for the detailed comments provided. In response, we reviewed the Abstract and the Results and Discussion sections. Where appropriate, we revised the wording to more clearly indicate when findings were derived from a single study and to ensure that interpretations accurately reflect the evidence base. We have also added minor wording/spelling corrections throughout the entire manuscript.

1. I appreciate that the authors generally use cautious language to avoid implying stronger causal relationships than the evidence supports. However, I suggest revising some statements in the Results, particularly where a finding is derived from a single study. Wording such as “One study reported…” may help clarify the source of the finding and prevent it from being interpreted as an overall conclusion of the review. For example, in Section 3.3.4, it may be clearer to write: “One study reported lower mortality and readmission rates… After adjustment, the difference…” This makes it explicit that these are findings reported by an individual study rather than interpretations drawn from the evidence.
I am not suggesting that this wording should be applied throughout the Results. In several places, the authors appropriately synthesise findings across studies. However, where the evidence remains uncertain, slightly more cautious wording could be considered. For example, in Section 3.3.3, “Evidence of possible deterioration included an increased TRIPS score (54% of transported infants) (ref), …” may be more appropriately cautious.

We thank the reviewer for this suggestion. We have revised the Results section to ensure that single-study findings are clearly indicated and not inadvertently interpreted as overall conclusions of the review. We incorporated more explicit wording regarding the origin of single-study findings to provide context for the reported results (see also our response to Comment 2).

2. In several places in the Discussion, findings from a single study appear to be presented as conclusions applying to the overall evidence base. For example, on page 17, lines 377–378, the authors state that “higher hospital readmission rates after discharge home were reported for transported infants compared to non-transported controls.” The subsequent discussion indicates that this finding was primarily derived from Lainwala et al., which included only extremely-low-birthweight

infants. When presented in the opening summary without this context, readers may interpret higher readmission as a consistent finding across the review.

We agree with the reviewer and have revised the Discussion accordingly. Throughout this section, and also in the Results, we have made changes to the wording to explicitly indicate the origin of findings where appropriate. Regarding the specific finding mentioned by the reviewer (readmission rates), we removed this statement from the opening summary of the Discussion section, as we agree that mentioning this finding without the appropriate context might lead to misinterpretations of the available evidence.

3. Page 18, line 401: a duplicated comma in this sentence.

Thank you. This has been corrected.

4. On page 18, lines 418–419, the authors state: “These findings may indicate that any transport-related instability is transient and its effects are limited to the immediate post-transport period.” I am uncertain whether this inference is supported by the available evidence. The absence of statistically significant differences in longer-term studies may reflect insufficient sample sizes, baseline differences between transported and non-transported infants, inadequate adjustment for confounding, or limited statistical precision. It therefore does not necessarily demonstrate that any instability is transient or restricted to the immediate post-transport period. I suggest that the authors reconsider or qualify this interpretation.

We agree that this interpretation is not supported by the available evidence. The sentence has been revised to avoid implying that the evidence demonstrates transient instability. In addition, we now place greater emphasis on limitations such as confounding, baseline differences between groups, and inadequate statistical adjustment where appropriate.

5. On page 19, line 440, the authors state that “parental distress emerged as a consistent and well-supported finding.” I suggest reconsidering whether “well-supported” appropriately reflects the strength of the evidence, as it may imply a high level of confidence. The number of studies was relatively limited, assessment timepoints varied substantially, and participants were predominantly white, married, college-educated mothers, which limits the applicability of the findings. Recurrence of stress or distress across studies does not establish its magnitude or demonstrate that transport itself caused the distress, particularly in the absence of an appropriate comparator.

We thank the reviewer for this important point. We agree that the term “well-supported” may be an overstatement. This wording has been revised throughout the manuscript. The Discussion now emphasises that descriptions of parental distress were recurrent across qualitative and quantitative studies, without implying a level of confidence that is not supported by the available evidence.

6. The comments above provide several examples rather than an exhaustive list. I encourage the authors to review the Discussion more broadly to ensure that causal interpretations and overall conclusions are proportionate to the observational, heterogeneous, and frequently single-study evidence.

We appreciate this recommendation and have conducted a broader review of the Discussion section. Throughout the manuscript, we have revised the wording used to describe findings, ensuring that conclusions remain proportionate to the available evidence.

7. The Abstract states that “this review is limited by substantial heterogeneity in outcome reporting, resulting in reliance on mostly unadjusted, single-study findings” Heterogeneity in outcome reporting

did not itself result in the included evidence being predominantly unadjusted or derived from single studies. These are separate, although related, limitations. I suggest presenting them in parallel, something like: “The evidence was limited by substantial heterogeneity in outcome reporting, predominantly unadjusted analyses, and findings that were frequently derived from single studies.”

We thank the reviewer for this suggestion and for proposing alternative wording. We agree and have revised the Abstract accordingly. The limitations are now presented in parallel, with the revised wording stating that the evidence was limited by substantial heterogeneity in outcome reporting, predominantly unadjusted analyses, and findings that were frequently derived from single studies.

VERSION 3 – REVIEW

**REVIEWER NAME**	Liu, Shiyao
**REVIEWER AFFILIATION**	University of Lancashire, School of Medicine
**REVIEWER CONFLICT OF INTEREST**	No Conflict of Interest
**AI DISCLOSURE**	No, I have not used AI
**DATE REVIEW RETURNED**	07-Sep-2026

**GENERAL COMMENTS**	The authors have addressed my comments, and I have no further concerns.

**REVIEWER NAME**	Haines, Charlotte
**REVIEWER AFFILIATION**	University Of Lancashire
**REVIEWER CONFLICT OF INTEREST**	No Conflict of Interest
**AI DISCLOSURE**	No, I have not used AI
**DATE REVIEW RETURNED**	06-Oct-2026

**GENERAL COMMENTS**	Thank you for resubmitting your amendments. I am happy that the outstanding comments have been addressed.
